# Radiotherapy for calcaneodynia, achillodynia, painful gonarthrosis, bursitis trochanterica, and painful shoulder syndrome - Early and late results of a prospective clinical quality assessment

**DOI:** 10.1186/s13014-018-1025-y

**Published:** 2018-04-19

**Authors:** Oliver Micke, Eyup Ugrak, Stefan Bartmann, Irenaeus A. Adamietz, Ulrich Schaefer, Rebecca Bueker, Klaus Kisters, M. Heinrich Seegenschmiedt, Khashayar Fakhrian, Ralph Muecke

**Affiliations:** 1 Department of Radiotherapy and Radiation Oncology, Franziskus Hospital Bielefeld, Kiskerstrasse 26, D-33615 Bielefeld, Germany; 2 Department of Internal Medicine, St. Anna Hospital, Herne, Germany; 3 Department of Urology, Lippe Hospital, Detmold, Germany; 4 Department of Radiotherapy and Radiation Oncology, Marien Hospital Herne, Ruhr University Bochum, Bochum, Germany; 5 Department of Radiotherapy, Lippe Hospital, Lemgo, Germany; 6 Center for Radiotherapy, Hamburg, Germany; 7 Radiotherapy RheinMainNahe, Bad Kreuznach, Germany

**Keywords:** Benign diseases, Radiotherapy, Calcaneodynia, Gonarthrosis, Bursitis trochanterica, Shoulder syndrome

## Abstract

**Background:**

The aim of this prospective clinical quality assessment was to evaluate the short-term and long-term efficacy of low dose radiotherapy (RT) for calcaneodynia, achillodynia, painful gonarthrosis, painful bursitis trochanterica, and painful shoulder syndrome.

**Methods:**

Between October 2011 and October 2013, patients with calcaneodynia, achillodynia, painful gonarthrosis, painful bursitis trochanterica, and painful shoulder syndrome were recruited for this prospective clinical quality assessment. Single doses of 0.5-1.0 Gy and a total dose of 6.0 Gy per series were used. Pain was measured before and directly after RT (early response) with a visual analogue scale (VAS). Additionally, pain relief was measured with the four-scale pain score according to “von Pannewitz” (VPS) immediately at the end of RT and during follow-up. Within this context we defined a good response as complete pain relief and markedly improved. The assessment of the long-term efficacy was carried out by a telephone survey.

**Results:**

703 evaluable patients (461 female, 242 male) with a mean age of 63.2 years (28-96) were recruited for this prospective clinical quality assessment. In 254 patients RT was performed with the linear accelerator, 449 patients received orthovoltage radiotherapy. After a median follow-up of 33 months (3-60) 437 patients could be reached for evaluation of follow up results. The mean VAS value before treatment was 6.63 (1.9-10) and immediately on completion of RT 4.51 (0-10) (*p* < 0,001). Concerning the VPS immediately on completion of RT, a good response could be achieved in 264/703 patients (37.6%), and with the follow up in 255/437 patients (58.4%) (*p* < 0.001). Only in patients with gonarthrosis we could not observe a significantly improved long-term success in comparison to the results immediately after RT (30.2% versus 29.9%).

**Conclusion:**

Low dose RT is a very effective treatment for the management of calcaneodynia, achillodynia, painful gonarthrosis, painful bursitis trochanterica, and painful shoulder syndrome. Due to the delayed onset of analgesic effects low dose RT results in a significantly improved long-term efficacy in comparison to the results immediately after RT particularly in patients with calcaneodynia, achillodynia, bursitis trochanterica, and shoulder syndrome.

## Background

There is a long tradition for low dose radiotherapy (RT) of painful benign skeletal diseases in Central Europe. RT of benign diseases accounts for about 8–10% of all RT procedures in Germany. This development of radiotherapy for benign disorders in the last years can be reasonably regarded as real renaissance.

As much as 70% of these indications represent painful disorders in the locomotor system [1–5].

Recent radiobiological experiments show that low doses of radiation have a modulatory activity on several inflammatory pathways and immune components like endothelial cells, mono- and polynuclear leukocytes and macrophages [6].

For this treatment, single doses of 0.5 to 1.0 Gy and total doses of 3.0 to 6.0 Gy per series are generally accepted.

The aim of this prospective clinical quality assessment was to analyse the therapeutic effect of low dose irradiation immediately after completion of RT and during follow-up and to identify possible prognostic factors in patients with calcaneodynia, achillodynia, painful gonarthrosis, painful shoulder syndrome and painful bursitis trochanterica. It is a well-known observation, that the pain relief after RT often occurs after a longer period of time, generally 6 to 12 weeks [1–3]. However, this phenomenon is not well described and possible implication are not known. Therefore, we examined with this prospective clinical quality assessment, whether a delayed onset of analgesic effects of RT leads to a significantly improved long-term success in comparison to the results immediately after RT.

## Methods

Between October 2011 and October 2013, patients with calcaneodynia, achillodynia, painful gonarthrosis, painful bursitis trochanterica, and painful shoulder syndrome were recruited for this prospective quality assessment. All patients had given their informed consent to the radiotherapy and to the participation in this prospective clinical quality assessment before enrolment.

RT was performed with both linear accelerator and orthovoltage conditions. Single doses of 0.5-1.0 Gy and a total dose of 6.0 Gy per series were used.

Pain was measured before and right after RT (early response) with a 10 scale visual analogue scale (VAS) (0 - no pain, 10 - strongest pain) [7]. Additionally, pain relief was measured with the four-scale pain score according to “von Pannewitz” (VPS) (complete pain relief, markedly improved, slightly improved, unchanged) immediately on completion of RT and during follow-up [8]. Within this context, we defined a ***good response*** as complete pain relief and markedly improved. The assessment of the long-term efficacy was carried out by a systematic telephone survey. The results were recorded in an Excel spreadsheet and then transferred to SPSS for evaluation after completion of the survey. A part of all treated patients were irradiated with a second series (*n* = 51), if there was no or only slight improvement after the first RT series. These results have been included in the evaluation.

### Statistics

All data were stored and analyzed using the SPSS statistical package 15.0 (SPSS Inc., Chicago, Illinois, USA). Descriptive statistics were computed for continuous and categorical variables. The statistics computed included mean and standard deviations of continuous variables, and frequencies and relative frequencies of categorical factors. Testing for differences in continuous and categorical variables within the groups was accomplished by the Wilcoxon Signed Rank Test. Testing for differences in continuous variables between the groups was accomplished by the Mann-Whitney U test, and in categorical variables between the groups with the Fisher’s Exact Test, as appropriate. All *P* values were two-sided statistical tests, and values of *P* < .05 were considered statistically significant.

## Results

### Patients

703 evaluable patients (461 female, 242 male) with a mean age of 63.3 years (28-96) were recruited for this prospective trial. The following diagnoses were given: 286 x calcaneodynia, 46 x achillodynia, 139 x gonarthrosis, 70 x bursitis trochanterica, and 162 x shoulder syndrome. Patient characteristics are given in Table 1.

**Table 1:** Patients and treatment characteristics

Diagnosis	Number	Mean Age (years)	Female/Male	Fractionation 12 × 0.5 Gy / 6 × 1.0 Gy	Technique Orthovolt / Linac	Median Follow up (months)
Calcaneodynia	286	56.8 (30-87)	219/67	265/21	284/2	34 (21-40)
Achillodynia	46	54.7 (28-76)	11/35	33/13	45/1	33 (23-39)
Gonarthrosis	139	70.9 (38-90)	78/61	112/27	53/86	19.5 (3-40)
Bursitis trochanterica	70	64.6 (43-88)	56/14	66/4	8/62	29 (3-39)
Shoulder Syndrome	162	69.5 (39-96)	97/65	120/42	56/106	42 (6-60)
All patients	703	63.2 (28-96)	461/242	596/107	446/257	33 (3-60)

### Treatment

In 254 patients RT was performed with the linear accelerator, 449 patients received orthovoltage radiotherapy. In 596 patients, RT was performed with 12 × 0.5 Gy, in 107 patients with 6 × 1.0 Gy. In 652 patients, RT was performed with one series, in 51 patients with two series in case of insufficient remission of pain after 3 months.

### Vas

The median VAS value before treatment was 7.0 (5-8) and immediately on completion of RT 4.5 (3-6) (*p* < 0,001). Results for the different diagnoses are given in Table 2.

**Table 2:** Median VAS-values before and immediately on completion of RT

Diagnosis	Median VAS value before RT (interquartile range)	Median VAS value immediately on completion of RT (interquartile range)	*P*-Value
Calcaneodynia	7.0 (5.425-8)	4.0 (2.5-6)	< 0.001
Achillodynia	6.0 (5-7.125)	4.0 (2-5)	< 0.001
Gonarthrosis	6.0 (5-8)	4.5 (3-6)	< 0.001
Bursitis trochanterica	7.0 (6-8)	5.0 (3.725-7.125)	< 0.001
Shoulder Syndrome	7.0 (5-8)	5.0 (3-6)	< 0.001
All patients	7.0 (5-8)	4.5 (3-6)	< 0.001

### VPS immediately on completion of RT

A total of 29 patients (4.1%) were free of pain, 234 (33.3%) were much improved, 233 (33.1%) reported slight improvement, and 207 (29.5%) experienced no change.

### VPS follow up

After a median follow-up of 33 months (3-60 months) 437 patients could be reached for evaluation of follow up results. 155 patients (35.5%) were free of pain, 100 (22.9%) had marked improvement, 65 (14.8%) had some improvement, and 117 (26.8%) experienced no change.

### Comparison of VPS

A good response immediately on completion of RT could be achieved in 264/703 patients (37.6%), and with the follow up in 255/437 patients (58.4%) (*p* < 0.001). Only in patients with gonarthrosis we could not observe an increase of good response (30.2% immediately on completion of RT versus 29.9% at the follow up time).

Results for the different diagnoses are given in Table 3.

**Table 3:** Good Response (%) immediately on completion of RT and during follow up

Diagnosis	Good Reponse on completion of RT	Good Reponse - Follow up	*P*-Value
Calcaneodynia	46.0% (131/286 patients)	80.7% (113/140 patients)	< 0.001
Achillodynia	39.1% (18/46 patients)	88.9% (24/27 patients)	=0.001
Gonarthrosis	30.9% (43/139 patients)	29.2% (33/113 patients)	=0.612
Bursitis trochanterica	27.1% (19/70 patients)	46.3% (31/67 patients)	=0.012
Shoulder Syndrome	32.7% (53/162 patients)	60% (54/90 patients)	< 0.001
All patients	37.6% (264/703 patients)	58.4% (255/437 patients)	< 0.001

### Comparison of results for the different disorders

Treatment results regarding the comparison between the different disorders are given in Table 4. In general, there was better effect of RT for the enthesiopathies in comparison with gonathrosis.

**Table 4:** Comparison between the diagnoses concerning Good Reponse on completion of RT, and Good Reponse - Follow up

Diagnosis	Good Reponse on completion of RT	*P*-Value	Good Reponse - Follow up	*P*-Value
Calcaneodynia	46.0%		80.7%	
Achillodynia	39.1%	=0.388	88.9%	=0.312
Calcaneodynia	46.0%		80.7%	
Gonarthrosis	30.9%	=0.003	29.2%	< 0.001
Calcaneodynia	46.0%		80.7%	
Bursitis trochanterica	27.1%	=0.004	46.3%	< 0.001
Calcaneodynia	46.0%		80.7%	
Shoulder Syndrome	32.7%	=0.006	60.7%	=0.001
Achillodynia	39.1%		88.9%	
Gonarthrosis	30.9%	=0.307	29.2%	< 0.001
Achillodynia	39.1%		88.9%	
Bursitis trochanterica	27.1%	=0.177	46.3%	< 0.001
Achillodynia	39.1%		88.9%	
Shoulder Syndrome	32.7%	=0.419	60.7%	=0.006
Gonarthrosis	30.9%		29.2%	
Bursitis trochanterica	27.1%	=0.572	46.3%	=0.021
Gonarthrosis	30.9%		29.2%	
Shoulder Syndrom	32.7%	=0.741	60.7%	< 0.001
Bursitis trochanterica	27.1%		46.3%	
Shoulder Syndrome	32.7%	=0.401	60.7%	=0.075

### Further results

Results regarding fractionation, number of series, and radiation unit (only for gonarthrosis, bursitis trochanterica, and shoulder syndrome) are given in Tables 5 and 6. Further significant differences between the groups were not found. No side effects have been observed.

**Table 5:** Influence of number of series, single dose, and gender on Good Reponse on completion of RT, and Good Reponse - Follow up

Parameter	Good Reponse on completion of RT	*P*-Value	Good Reponse - Follow up	*P*-Value
One series (*n* = 652)	38.4%		58.8%	
Two series (*n* = 51)	27.5%	=0.102	55.3%	=0.673
12 × 0.5 Gy (*n* = 596)	38.6%		58.5%	
6 × 1.0 Gy (*n* = 107)	32.7%	=0.25	58.6%	=0.982
Female (*n* = 461)	38.1%		55.2%	
Male (*n* = 242)	36.8%	=0.726	64.1%	=0.071

**Table 6:** Influence of radiation treatment unit on Good Reponse on completion of RT, and Good Reponse - Follow up for gonarthrosis, bursitis trochanterica, and shoulder syndrome

Parameter	Good Reponse on completion of RT	*P*-Value	Good Reponse - Follow up	*P*-Value
Gonarthrosis				
Linac (*n* = 86)	31.4%		32.8%	
Orthovolt (*n* = 53)	30.1%	=0.882	21.6%	=0.218
Bursitis trochanterica				
Linac (*n* = 62)	29.0%		44.1%	
Orthovolt (*n* = 8)	12.5%	=0.326	62.5%	=0.330
Shoulder Syndrome				
Linac (*n* = 106)	27.4%		57.4%	
Orthovolt (*n* = 56)	42.8%	=0.046	67.8%	=0.350

## Discussion

The above shown results of our prospective clinical quality assessment confirm the results of recently published retrospective and prospective randomized studies with a good analgesic effect of low dose radiotherapy for patients with calcaneodynia, achillodynia, painful gonarthrosis, painful bursitis trochanterica, and painful shoulder syndrome [9–23].

The precise pathophysiological mechanisms of pain relief after RT are still not well defined. Recent radiobiological experiments show that low doses of radiation have an anti-inflammatory efficacy based on the modulation of a multitude of inflammatory pathways and cellular components. This includes immune components like endothelial cells, mono- and polynuclear leukocytes and macrophages, and an influence on the vascular endothelium with improved tissue perfusion, destruction of inflammatory cells (in particular lymphocytes) with release of cytokines and proteolytic enzymes, modulation of the vegetative nervous system, altering of the tissue pH and increased membrane permeability. Most likely, irradiation does not act through a single mechanism but through a complex interaction of different effects [6].

We observed a significantly improved long-term efficacy in comparison to the results immediately after RT in patients with calcaneodynia, achillodynia, bursitis trochanterica, and shoulder syndrome. This could be due to the delayed clinical onset of effects, which is most likely due to the also delayed onset of above mentioned radiobiological mechanisms. However, the group of patients with gonarthrosis was the only one without this observed delayed effect. Most likely, with low dose RT we can achieve more complete remissions in patients with calcaneodynia, achillodynia, bursitis trochanterica, and shoulder syndrome. In contrast, gonarthrosis is described to be an irreversible pathological process, with cartilaginous and bony destructions, which cannot reversed by radiotherapy. These irreversible destructions initiate a variety inflammatory processes leading to pain, swelling etc. under the clinical picture of activated osteoarthritis [8, 9]. Here, low doses RT may be helpful by alleviation of inflammation and pain in these acute episodes of this chronic joint disorders. However, the underlying pathophysiological problem remains more or less unchanged by RT, even so RT can arrest and slow down the progressive joint destruction in osteoarthritis by the anti-inflammatory effect of low dose RT. Therefore, the analgesic effect is only moderate compared to other indications, in particular the enthesiopathies [1, 4, 8].

Clearly, a possible placebo effect of low dose RT for pain treatment cannot completely be excluded. In previously published early double-blinded studies from the 1970s, a large variety of different degenerative skeletal diseases were treated with low-dose RT. These studies could not prove a significantly higher response for the RT group in comparison to the placebo group [24–26].

Radiation side effects did not occur in any of our patients. This corresponds to the reported absence of chronic or acute adverse effects in the literature [9–23].

## Conclusion

Low dose RT is a very effective treatment for the management of calcaneodynia, achillodynia, painful gonarthrosis, painful bursitis trochanterica, and painful shoulder syndrome. Due to the delayed onset of analgesic effects low dose RT results in a significantly improved long-term efficacy in comparison to the results immediately after RT particularly in patients with calcaneodynia, achillodynia, bursitis trochanterica, and shoulder syndrome.

## Abbreviations

Gy: Gray
RT: Radiotherapy
VAS: Visual analogue scale
VPS: von Pannewitz Score
